# Copy number loss upstream of *RAI1* uncovers gene expression regulatory region that may impact Potocki–Lupski syndrome diagnosis

**DOI:** 10.1186/s13039-015-0179-7

**Published:** 2015-10-05

**Authors:** Joseph T. Alaimo, Sureni V. Mullegama, Mary Ann Thomas, Sarah H. Elsea

**Affiliations:** Department of Molecular and Human Genetics, Baylor College of Medicine, One Baylor Plaza, NAB2015, Houston, TX 77030 USA; Department of Medical Genetics, Alberta Children’s Hospital, University of Calgary, Calgary, AB CA T2N 1N4 Canada

**Keywords:** *RAI1*, Structural variation, Copy number variation, SMS, PTLS, ENCODE

## Abstract

The identification of structural variants of uncertain clinical significance is increasing; however, studies delineating the functional consequence of these variants in the pathogenicity of phenotypic features are lacking. Understanding the consequence of structural variants such as copy number alterations and their role in gene expression changes is paramount in order to perform a comprehensive analysis of genetic effects on phenotypic variation and disease. *RAI1* is a dosage-sensitive essential neurodevelopmental gene. Copy number loss of *RAI1* results in Smith-Magenis syndrome while copy number gain results in Potocki-Lupski syndrome. Here, we present a case of a six year old female with a newly identified maternally inherited copy number loss that lies within the Smith-Magenis syndrome common deletion region, but *RAI1* copy number is normal. Integration of the Encyclopedia of DNA Elements (ENCODE) data at the affected region suggests that the deletion disrupts several cis-acting regulatory elements upstream of *RAI1,* such as multiple repressor sites and an insulator region. Gene expression studies revealed that both the proband and the mother have significantly elevated *RAI1* mRNA levels suggesting that the structural variant alters gene expression regulation. The proband and the mother both have some features of Potocki-Lupski syndrome, while the child appears to be more affected with autistic-like features. Overall, our work demonstrates that the integration of ENCODE data with structural variants of uncertain significance aids in delineating a functional consequence to a genomic aberration and subsequent diagnosis.

## Background

Chromosomal microarrays (CMA) have vastly changed the identification of structural variants across the genome. CMA is considered a first-tier clinical diagnostic test for individuals with idiopathic developmental delay, intellectual disability, autism spectrum disorders, and multiple congenital anomalies [1–4]. Determining the clinical significance of variants identified through CMA is challenging and requires the integration of information across laboratories and experimental evidence presented in the literature. Furthermore, the identification of novel structural variants confers an even greater challenge because the pathogenicity of the mutation is unknown primarily due to the lack of molecular and functional studies required to implicate a genotype/phenotype correlation.

The chromosomal locus 17p11.2 is an error prone region that frequently undergoes recombination events resulting in genomic rearrangements. Within this region lies retinoic acid-induced 1 (*RAI1*), a dosage-sensitive gene responsible in the pathogenicity of Smith-Magenis syndrome (SMS, MIM 182290) when haploinsufficient and Potocki-Lupski syndrome (PTLS, MIM 610883) when duplicated [5]. SMS is characterized by a distinct facial gestalt, variable intellectual disability, early-onset of obesity, inverted sleeping patterns, and a wide range of behavioral problems including self-injury and stereotypies [5]. PTLS phenotypic features are reportedly less comprehensibly characterized and much milder than what is observed in SMS, but most affected individuals present with variable intellectual disability, autistic-like features, cardiovascular malformations, and sleep apnea [6–8].

*RAI1* is an essential neurodevelopmental gene that requires tightly controlled regulation of expression temporally and spatially [9], but the genetic landscape encompassing the regulation of *RAI1* gene expression has largely been unexplored. Here, we characterized the role of a newly identified maternally inherited deletion proximal to *RAI1* relative to *RAI1* gene expression and uncover, through the use of the Encyclopedia of DNA Elements (ENCODE), the *cis-*regulatory regions that are compromised, assigning functional consequence to the genomic aberration.

## Case presentation

**Fig. 1:**
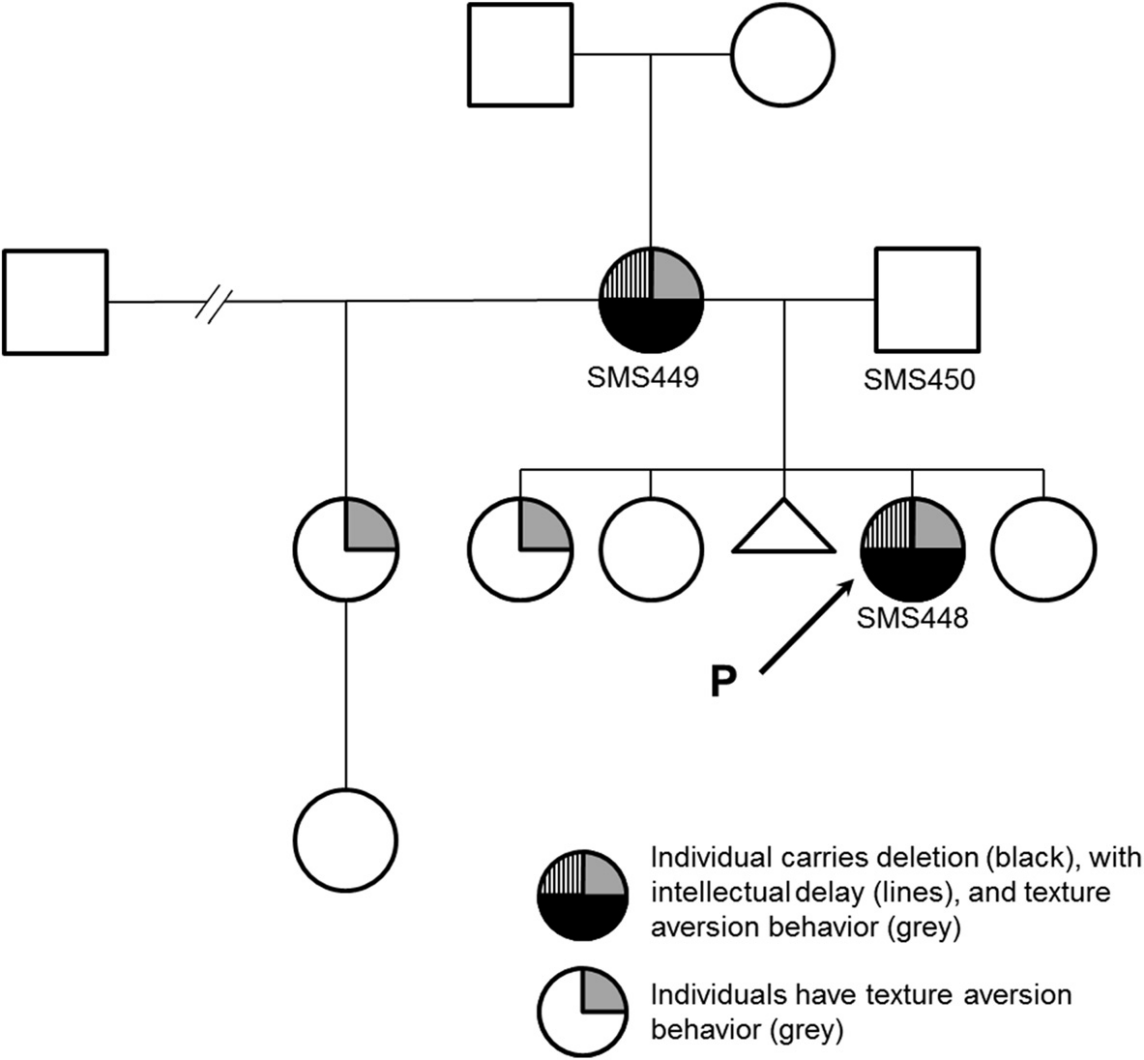
Pedigree of family with 17p11.2 deletion. The proband (SMS448) is denoted by arrow and carries a maternally inherited 17p11.2 deletion (black semicircle) with intellectual delay (vertical lines) and severe texture aversion behavior (grey). A full-sibling and a half-sibling also display severe texture aversion behavior (grey)

**Fig. 2:**
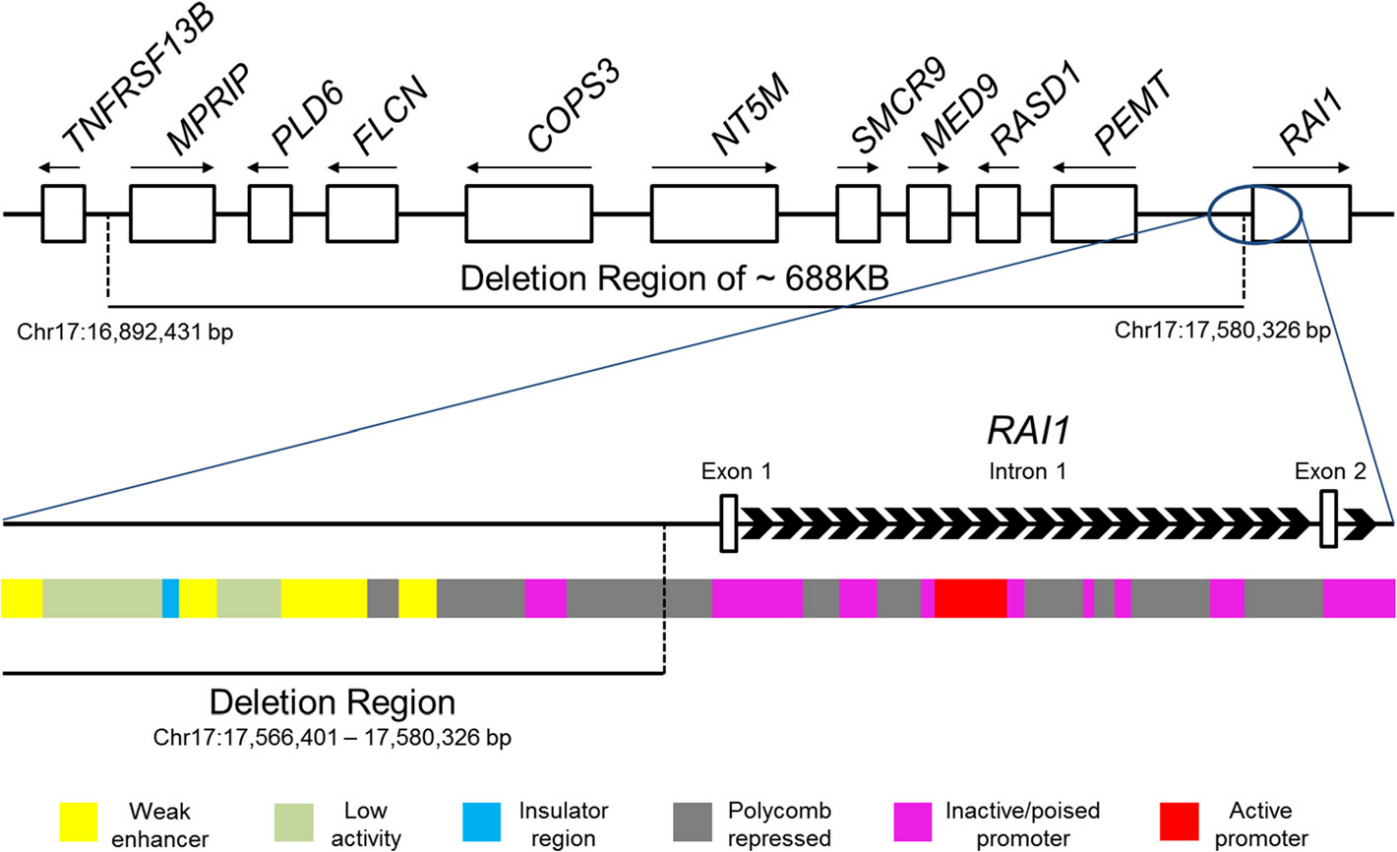
Genomic structure of 17p11.2 CNV and ChromHMM ENCODE regulatory sites. (Top) The maternally inherited deletion spans a maximum of about 688 kb and encompasses 9 genes (white). Arrows represent gene orientation. (Bottom) Enlarged region of deletion shows that the maximum distal breakpoint leaves *RAI1* coding regions (white) intact. ChromHMM annotated regulatory sites within H1-hESC from ENCODE are labeled directly underneath. Red = active promoter including transcriptional start site, pink = inactive promoter, dark grey = polycomb repressed, yellow = weak enhancer, light green = low activity sites, and blue = insulator sites

A female proband (SMS448) came to our attention at the age of six years because of behavioral problems and global developmental delay. She was born to unrelated parents of maternal Flemish, Norwegian, and Scandinavian descent and paternal Scottish and English descent. She had an unremarkable prenatal history with no reported maternal illness and was delivered at term with no complications or need for resuscitation. At the time of the visit, her height was between 50–75^th^ percentile, weight was at 97^th^ percentile and head circumference was at 50^th^ percentile. She presented with several developmental issues including motor delay with associated decreased strength, speech delay for which she received speech therapy from 18 months to 3 years of age, sensory integration disorder as diagnosed by occupational therapy, socialization issues, sleep disturbance and repeated aggressive behavior when frustrated. Physical examination revealed a square face, synophrys, deep-set eyes, upslanting palpebral fissures, a prominent nasal root and smooth philtrum. She was missing primary and adult lateral incisors, had increased spacing between lower probable central incisors, widely spaced nipples and thin nails. A review at 9 years of age revealed improvement in her development and behavior; however, she frequently exhibited withdrawal or attention problems. She repeated kindergarten but at this visit, was attending a regular grade 3 class with no aide or extra support. Her growth pattern and physical features remained consistent as her first visit. The proband was referred for a possible diagnosis of Smith-Magenis syndrome based upon abnormal array-CGH findings using CytoChip™ ISCA 8x60K v2.0 (BlueGnome). The DNA microarray analysis was performed in a clinical cytogenetic laboratory and the reported minimal coordinates for the abnormality were arr[hg19] 17p11.2p11.2 (16,936,632–17,509,926)×1 and the reported maximal coordinates for the abnormality were arr[hg19] 17p11.2p11.2 (16,892,431–17,580,326)×1. Assessment of the origin of the deletion by fluorescence *in situ* hybridization (FISH) revealed a maternally derived deletion (SMS449). The paternal FISH result was normal (data not shown). Siblings and maternal grandparents of SMS448 were not tested, and it is not known whether any harbor the copy number variant (CNV) (Fig. 1). This deletion region lies within the SMS common interval and encompasses nine genes: *MPRIP*, *PLD6*, *FLCN*, *COPS3*, *NT5M*, *SMCR9*, *MED9*, *RASD1* and *PEMT* and also overlaps with the region for the dominant genetic disorder, Birt-Hogg-Dubé syndrome (BHDS, MIM 135150) (Fig. 2, top). BHDS typically features fibrofolliculomas, bilateral lung cysts, history of spontaneous pneumothorax, and bilateral and multifocal renal tumors, but family history was negative for any of these features. Individuals with SMS who carry the common deletion have a deletion of *FLCN,* and to date, no reports of BHDS have been documented. An SMS individual with a history of 3 spontaneous pneumothoraces has been reported (but no other features of BHDS); however, sequence analysis revealed a missense mutation in *FLCN* affecting transcript variant 2 [10]. There are no reports of spontaneous pneumothorax or other features of BHDS in PTLS patients. Furthermore, the other genes within the deletion region have not been found to be associated with other genetic disorders.

It is unclear whether such a deletion could be deleterious and manifest the phenotype present in this child. However, the proband’s mother carries this deletion and has reported learning difficulties and significant sensory issues. Furthermore, other siblings of the proband, including a full and maternal half sibling have a similar hypersensitivity to textures and texture aversion behavior, although they have not been assessed for the CNV (Fig. 1). Phenotypic comparison of the proband to SMS revealed some similarities, such as the motor and speech delay, sensory issues, and increased weight. However, the proximal breakpoint of the deletion falls distal to *PEMT* but proximal to *RAI1,* resulting in normal copy number of the causative gene for SMS. Reduced *RAI1* mRNA expression is reported in SMS patients and can serve as an additional molecular diagnostic indicator for the disorder [5]. Therefore, we evaluated whether the deletion may affect *RAI1* gene expression on peripheral blood samples obtained from the proband, mother, father, and normal controls.

## Results and discussion

**Fig. 3:**
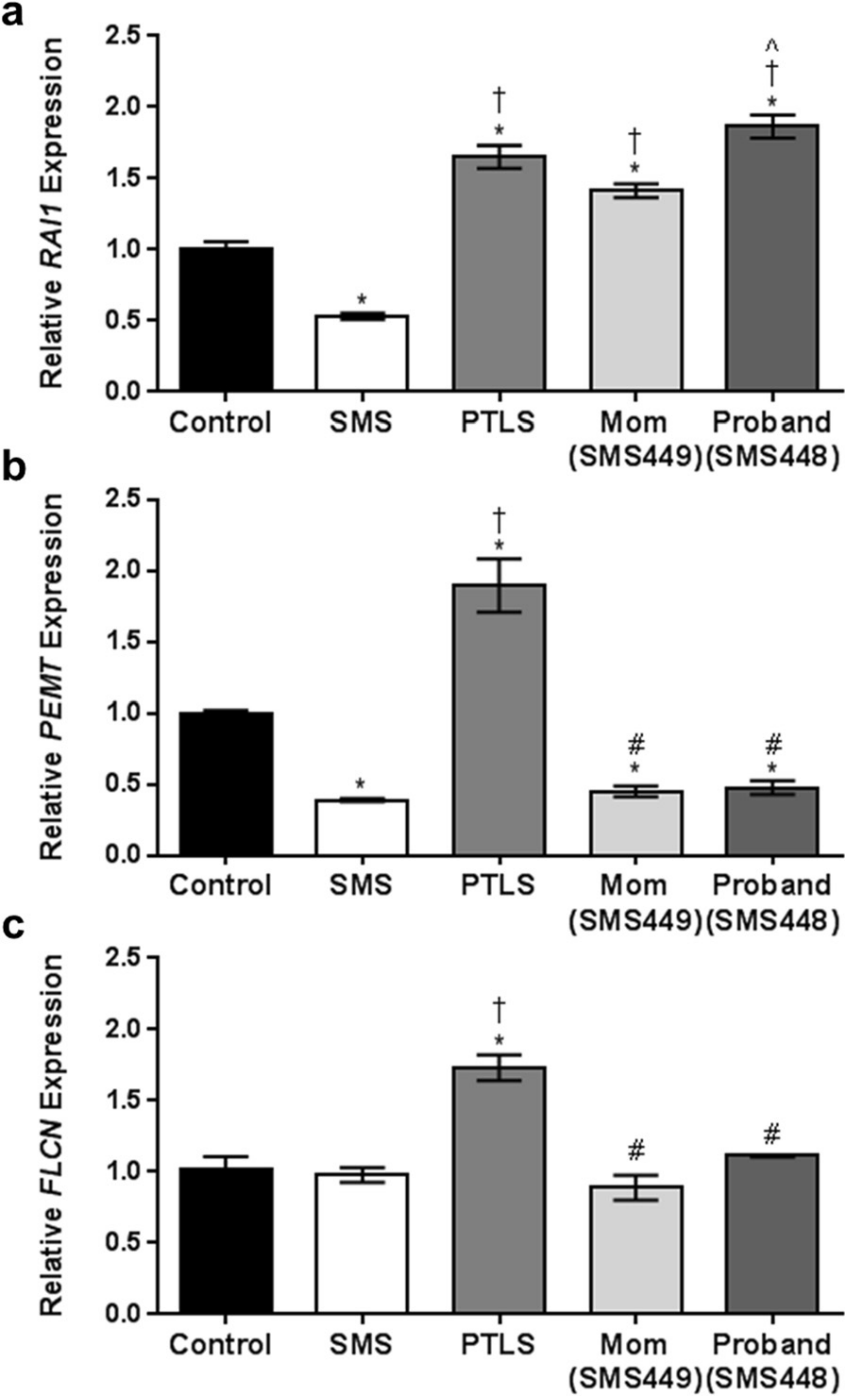
RT-qPCR expression values of genes within and proximal to 17p11.2 deletion in SMS449 and SMS448. (**a**) *RAI1* gene expression values are significantly reduced in SMS and significantly elevated in PTLS. Both the mother (SMS449) and the proband (SMS448) have significantly elevated *RAI1* mRNA levels relative to clinically normal controls, while SMS448 also has significantly elevated levels relative to SMS449. (**b**) *PEMT* in both SMS449 and SMS448 have significantly reduced mRNA levels, similar to other individuals with SMS deletions. *PEMT* levels in individuals with PTLS are significantly elevated (**c**) *FLCN* levels are not compromised in either SMS449 and SMS448 despite only having one copy. Results are similar to other SMS deletions. Individuals with PTLS have significantly elevated levels. Data are plotted as means +/-SEM. *p < 0.05 (relative to control) ^†^ *p* < 0.05 (relative to SMS), ^#^ *p* < 0.05 (relative to PTLS), ^*p* < 0.05 (relative to SMS449)

In order to establish *RAI1* expression levels for comparison to our patient, we measured *RAI1* mRNA levels in lymphoblast cell lines from individuals with SMS and PTLS. As expected, *RAI1* mRNA levels were significantly reduced in SMS samples (*p* = 0.0006) and overexpressed in samples from individuals with PTLS (*p* < 0.0001) (Fig. 3a). Interestingly, we observed that the proband (SMS448) had a significant increase in *RAI1* mRNA levels of ~1.9 fold (*p* < 0.0001), while maternal (SMS449) expression levels were also significantly elevated to ~1.4 fold relative to controls (*p* < 0.0001) (Fig. 3a). The paternal *RAI1* mRNA level was not significantly different relative to other controls and therefore, was used as a control sample (data not shown). Next, we tested the expression of two other genes in the deletion region to confirm that reduction in gene dosage does alter mRNA levels. First, we tested *PEMT* in both SMS448 and SMS449 and found significantly reduced mRNA levels relative to normal controls (SMS448; *p* = 0.0434, SMS449 *p* = 0.0322) (Fig. 3b). The reduction of *PEMT* mRNA levels was also observed in individuals with SMS (*p* = 0.0026) but elevated in individuals with PTLS (*p* < 0.0001) (Fig. 3b). We also tested the expression level of *FLCN* and surprisingly found that the mRNA levels were not significantly compromised relative to normal controls in either SMS448 or SMS449 (Fig. 3c). Similarly, for SMS samples, we observed no expression changes in *FLCN,* despite copy number loss, while a significant increase in mRNA levels was found in PTLS samples (*p* < 0.0001) (Fig. 3c). Given the *FLCN* expression data and the lack of clinical findings in both mother and child, BHDS is not likely a medical concern. In summary, expression studies indicate that this particular deletion region increases *RAI1* gene expression, which may lead to some features observed in the proband and her mother. However, the mechanism of altered *RAI1* gene regulation is unclear.

In order to further explore the possible impact of this chromosomal deletion on the patient phenotype, we surveyed the Database of Genomic Variants (DGV) and found no deletions with identical coordinates to what is observed in SMS448 and SMS449. Within the deletion coordinates there are 33 reported deletions, while another spans the proximal breakpoint and another spans distal breakpoints and includes *RAI1*. Since the CNV breakpoints may disrupt the genetic structure of *RAI1* transcriptional regulatory elements, we utilized ENCODE data to uncover how the regulatory landscape may be altered. ENCODE includes data for the identification and quantification of RNA molecules in whole cells and sub-cellular compartments, mapping of protein-coding regions, chromatin and DNA accessibility, mapping of histone modifications and transcription binding sites, and DNA methylation status [11]. To uncover the putative regulatory region of *RAI1*, we specifically focused on the chromatin state segmentation analysis which includes the integration of ENCODE ChIP-seq, DNAse-seq, and FAIRE-seq data using a Hidden Markov Model (termed ChromHMM) in human embryonic stem cells (hESC) [12]. Interestingly, we found a variety of regulatory elements within the *RAI1* coding sequence and upstream of the transcriptional start site (Fig. 2, bottom). *RAI1* has two non-coding exons, and ChromHMM revealed two active promoter sites within H1-hESC; one located within intron one (Fig. 2, bottom, in red) and another in intron two (data not shown). Furthermore, the first active promoter region is surrounded by repressor regions (dark grey) and inactive but poised promoters (pink) (Fig. 2, bottom). Recent studies have shown that it is common for important developmental genes especially in hESC to have a mixture of active and poised promoter regions. It is thought that such a mixture serves the purpose for achieving alternative developmental cell fates or lineages [13]. The distal breakpoint of the CNV leaves most of the promoter regions intact except for one region (Fig. 2, Bottom). In addition, the deletion region also alters the regulatory structure of repressor (dark grey), enhancer (yellow) and insulator sites (blue) upstream of the *RAI1* transcriptional start site (Fig. 2, bottom).

The net effect of such a loss in terms of *RAI1* expression is unclear. The ENCODE data suggest that due to its position within the regulatory landscape, the annotated insulator site is likely blocking any upstream cis-acting enhancer site activity. However, the enhance regions are “weak” due to the low detection of histone H3 lysine 4 methylation within this region, suggesting minimal impact on expression. The abundant detection of histone H3 lysine 27 tri-methylation, particularly around the promoter region, provides strong evidence of polycomb-dependent gene repression. Therefore, it is not surprising to observe significantly elevated gene expression values of *RAI1* in both SMS448 and SMS449 based on this analysis (Fig. 3a). There may be other mechanisms that are contributing to the overexpression of *RAI1*. The genes that reside within the genomic loss may lead to global transcriptome changes which may include *RAI1* itself or another gene involved in regulating *RAI1.*

Some of the phenotypic features of SMS448 in conjunction with gene expression data are suggestive of PTLS. Previous studies have identified the smallest region of overlap of 17p11.2 duplications causing PTLS. In this particular study, three patients with < 1 Mb duplication did not exhibit a phenotype distinct from PTLS narrowing the genomic interval to include solely *RAI1* and surrounding regulatory sequences [8]. Examination of SMS448 relative to these individuals revealed overlapping features including normal growth measurements, global developmental delay, intellectual delay, language impairment, early behavioral issues with socialization and aggression, sleep problems, sensory integration disorder, dysmorphic facial features, and dental abnormalities. Interestingly, two of the three individuals initially displayed poor scholastic performance requiring aid, but were later enrolled in regular education classes similar to SMS448. However, some of the other phenotypic hallmarks of PTLS such as poor feeding as an infant, failure to thrive and hypotonia as an infant were absent from SMS448. Although, of the three only one individual displayed all three features while another only displayed hypotonia as an infant. This information was unknown for the third individual [8]. Therefore, *RAI1* overexpression may be contributing to these features in SMS448. Interestingly, there are also reported cases describing families of inherited PTLS, all of which are due to maternal transmission, and suggests that *RAI1* overexpression is more tolerated than haploinsufficiency [7, 14].

It is also interesting to note that the proband (SMS448) has higher *RAI1* mRNA levels than what is observed in her mother, SMS449 (*p* = 0.0003) (Fig. 3a). One possible rationalization could be that SMS448 may harbor another mutation in a gene that regulates *RAI1* gene expression, but further molecular analysis is required. The other phenotypic features such as wide spaced nipples, absence of both primary and secondary lateral incisors, and fine hair and nails have not been reported in PTLS and may potentially indicate the role of an additional gene important for the development of tissues arising from the ectoderm. There are a wide range of ectodermal dysplasia disorders that have mild to severe symptoms, but further investigation is required. These additional phenotypic features may also be the consequence of stochastic genome alterations directly impacting gene expression and function [15].

## Conclusions

In conclusion, we present a patient that carries a maternally inherited deletion that results in significantly elevated *RAI1* mRNA levels,which likely contribute to the overall phenotype including global developmental delay, behavior, and dysmorphisms in this patient. Since no other CNV was identified, additional testing such as whole-exome sequencing will be required to determine if the phenotypic features observed in this patient are the result of mutation at more than one locus. Furthermore, future work utilizing and integrating ENCODE data to infer function from newly reported genomic structural variants may provide clinicians and researchers an improved method for diagnosis.

## Methods

### Gene expression analysis

Peripheral blood samples were collected from SMS448 (proband), SMS449 (mother), SMS450 (father) and clinically normal controls. In addition, we used patient lymphoblast cell lines from Smith-Magenis syndrome (SMS129, SMS123, SMS105), Potocki-Lupski syndrome (SMS224 and GM23053) and controls (GM23054, GM23055, GM22677). GM23053, GM23054, GM23055, GM22677 were created by Coriell Cell Repositories (Camden, NJ, USA). Total RNA was isolated according to standard methods (Invitrogen, Carlsbad, CA), quantified using the NanoDrop® ND-100 Spectrophotometer, and reverse transcribed with qSCRIPT cDNA SuperMix (Quanta Biosciences, Inc., Gaithersburg, MD) according to the manufacturer’s instructions. Real-time quantitative PCR (RT-qPCR) was performed as previously described [16]. Briefly, Taqman probes (Life Technologies, Carlsbad, CA) for *RAI1* (Hs01554690_m1), *FLCN* (Hs00376065_m1), *PEMT* (Hs00540979_m1), and *GAPDH* (Hs9999905_m1) were used. *GAPDH* was used as an endogenous control, and all cDNA samples were run in triplicate in 10 uL reaction volumes and analyzed using the BioRad CFX Connect™ Real-Time PCR Detection System. Results are expressed as fold-change relative to normal controls, including the clinically normal father (SMS450). Multiple comparisons were performed using an ANOVA and Tukey’s post-hoc correction. Statistical significance was determined at *P* < 0.05.

### Bioinformatics

Array CGH analysis identified a copy number loss on the short arm of chromosome 17 with the following basepair coordinates; minimum, arr[hg18] 17p11.2p11.2 (16,877,357–17,450,651)×1 and maximum, arr[hg18] 17p11.2p11.2 (16,833,156–17,521,051)×1. Using the batch coordinate conversion tool in Genome Browser (LiftOver: https://genome.ucsc.edu/cgi-bin/hgLiftOver), both coordinates were converted to GRCh37/hg19 resulting in minimum deletion coordinates of chr17:16,936,632–17,509,926 and maximum deletion coordinates of chr17:16,892,431–17,580,326. We used the maximum coordinates for the evaluation of the ENCODE data.

## Consent

The Institutional Review Board at Baylor College of Medicine approved this study. Written informed consent was obtained from the patient and her parents for publication of this case report. A copy of the written consent is available for review by the Editor-in-Chief of this journal.

## Abbreviations

CMA: Chromosomal microarray
SMS: Smith-Magenis syndrome
PTLS: Potocki-Lupski syndrome
CNV: Copy number variant
ENCODE: Encyclopedia of DNA elements
CGH: Comparative genomic hybridization
Mb: Megabase
DGV: Database of Genomic Variants
ChromHMM: Chromatin state segmentation Hidden Markov Model
hESC: Human embryonic stem cell
